# Mechanics Characteristics of a 3D Star-Shaped Negative Poisson’s Ratio Composite Structure

**DOI:** 10.3390/ma16113950

**Published:** 2023-05-25

**Authors:** Linyi Yang, Mao Ye, Yonghui Huang, Jingkun Dong

**Affiliations:** Wind Engineering and Engineering Vibration Research Center, Guangzhou University, Guangzhou 510006, China

**Keywords:** honeycomb structure, negative Poisson’s ratio, numerical analysis, experimental study

## Abstract

A negative Poisson’s ratio honeycomb material has the characteristics of anti-conventional deformation behavior and high impact resistance, which is a new lightweight microstructure material with broad application prospects. However, most of the current research is still at the microscopic level and two-dimensional level, and little research has been carried out for three-dimensional structures. Compared with the two-dimensional level, three-dimensional negative Poisson’s ratio structural mechanics metamaterials have the advantages of a lighter mass, higher material utilization, and more stable mechanical properties, and they have great potential for development in the fields of aerospace, the defense industry, and vehicles and ships. This paper presents a novel 3D star-shaped negative Poisson’s ratio cell and composite structure, inspired by the octagon-shaped 2D negative Poisson’s ratio cell. The article carried out a model experimental study with the help of 3D printing technology and compared it with the numerical simulation results. The effects of structural form and material properties on the mechanical characteristics of 3D star-shaped negative Poisson’s ratio composite structures were investigated through a parametric analysis system. The results show that the error of the equivalent elastic modulus and the equivalent Poisson’s ratio of the 3D negative Poisson’s ratio cell and the composite structure is within 5%. The authors found that the size of the cell structure is the main factor affecting the equivalent Poisson’s ratio and the equivalent elastic modulus of the star-shaped 3D negative Poisson’s ratio composite structure. Furthermore, among the eight real materials tested, rubber exhibited the best negative Poisson’s ratio effect, while the copper alloy showed the best effect among the metal materials, with a Poisson’s ratio between −0.058 to −0.050.

## 1. Introduction

Under uniaxial tensile loads, conventional materials usually experience a decrease in their cross-sectional area perpendicular to the direction of the applied load, demonstrating positive Poisson’s ratio behavior. Negative Poisson’s ratio materials demonstrate a unique behavior when subjected to uniaxial tensile loads, expanding in the direction perpendicular to the applied force. This phenomenon is commonly known as the negative Poisson’s ratio effect [1]. This unique tensile expansion behavior makes the negative Poisson’s ratio have the advantages of being light weight and having high shear modulus, excellent impact resistance, good energy absorption and vibration, and noise reduction capabilities [2], and it has broad application prospects in many fields [3]. In aerospace engineering, Poisson’s ratio can be used to design metamaterials as in-orbit satellite launch surface materials, which can improve the deformation ability of in-orbit satellite antenna reflective surface reconstruction processes, reduce local stress and actuation force, and improve the accuracy of surface reconstruction [4]. In biomedical engineering, the use of 2D negative Poisson’s ratio materials to make auxiliary tubular structures can significantly increase the oxygen-aiding function of oxygen-enhancing tube structures [5]. Chen et al. [6] investigated the effect of negative Poisson’s ratio bolts on rock reinforcement using RFPA software. The experimental results proved that the negative Poisson’s ratio anchor can significantly improve the bearing capacity of anchored rock and absorb more energy. Wu et al. [7] proposed a double concave lens-shaped structure with a large value of negative Poisson’s ratio (LNPR) and designed a new vibration isolator with frequency-dependent damping characteristics. The negative Poisson’s ratio structure has evolved and developed into a concave-angle structure [8,9,10], a chiral structure [11], a rotating rigid body structure [12], a perforated plate structure [13], a pleated structure [14], and other structural forms. The concave angle structure can be divided into an arrow [9], a hexagon [8], and a star [10] according to the number.

The deformation characteristics and mechanical properties of honeycomb structures under loads such as tensile, bending, and torsion are also hot topics of research. Li et al. [15] demonstrated through static dynamic experimental tests that negative Poisson’s ratio composites have higher indentation stiffness and impact resistance compared with ordinary composites. Tho et al. [16] used the third-order shear deformation theory combined with the phase field theory to investigate core layer fracture only. The free vibration response and static bending of laminated composite plates with only core fracture were modeled. Zhang et al. [17] investigated the dynamic response of honeycomb sandwich panels under different loads, such as step load, wind-burst load, sinusoidal load, triangular load, and incremental load and verified the excellent mechanical properties of the negative Poisson’s ratio material. Based on finite element analysis, Zhu et al. [18] developed a novel reinforced six-arm missing pillar chiral tensile expansion metamaterial with an adjustable constant negative Poisson’s ratio in a large deformation range, revealing the microstructure–mechanical property relationship. However, the conventional negative Poisson’s ratio honeycomb is mostly a two-dimensional structure and less studied for three dimensions [19]. This traditional two-dimensional honeycomb model exhibits a negative Poisson’s ratio effect when loaded in-plane but does not show the pulling and swelling effect when loaded out-of-plane [20]. The research on negative Poisson’s ratio materials is still focused on theoretical studies and is limited in application.

Three-dimensional printing technology has matured and introduced new design dimensions, enriching the diversity of structural design and making negative Poisson’s ratio structures with more excellent properties. Jiang et al. [21] used 3D printing technology to print a modified “Buckley crystal assisted structure” and demonstrated the ability of this new implant to contract laterally under compression, which can be used to relieve lumbar disc herniation. Kim et al. [22] developed new soft-assisted structures with the help of 3D printing technology to achieve high manufacturing reliability and repeatability. Xue et al. [23] investigated the properties of negative Poisson’s ratio and folded hexagonal honeycomb structures through light-curing 3D printing experiments.

To this end, this paper proposes a three-dimensional negative Poisson’s ratio cell and a corresponding composite structure based on a two-dimensional star-shaped negative Poisson’s ratio cell. This structure has negative Poisson’s ratio properties in all three main directions and is lighter than the two-dimensional structure. With the improvement in processing and the preparation process, it will also have a broader application prospect. The authors firstly used the finite element method to simulate the star-shaped negative Poisson’s ratio structure numerically, then carried out a model compression test with the help of 3D printing technology, and finally studied the influence of the structural form and material properties on the mechanical characteristics of the 3D star-shaped negative Poisson’s ratio composite structure through a parametric analysis system.

## 2. Materials and Methods

### 2.1. Three-Dimensional Star-Shaped Negative Poisson’s Ratio Structure

**Figure 1:**
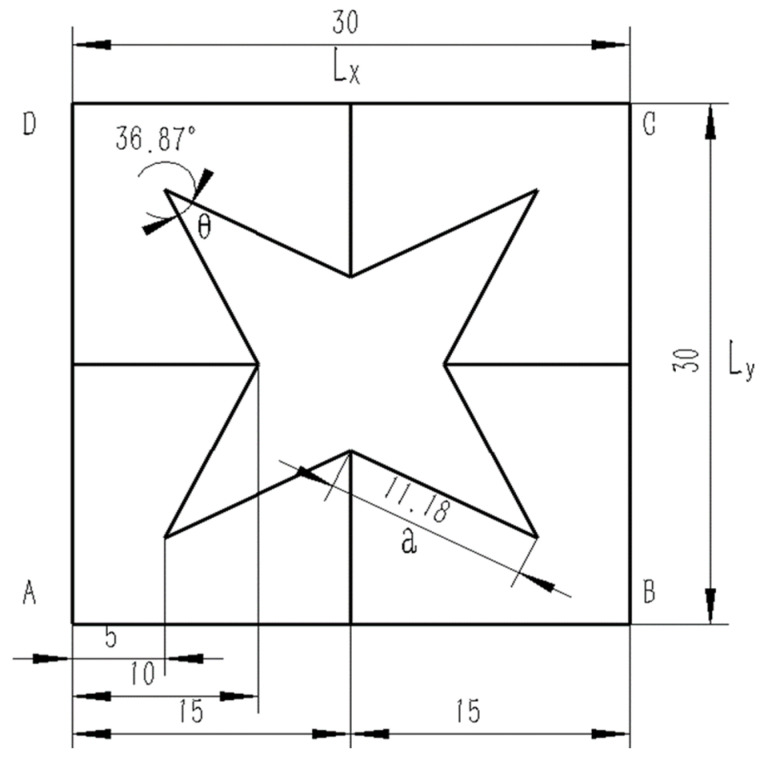
Structural geometric proportions and dimensions.

**Figure 2:**
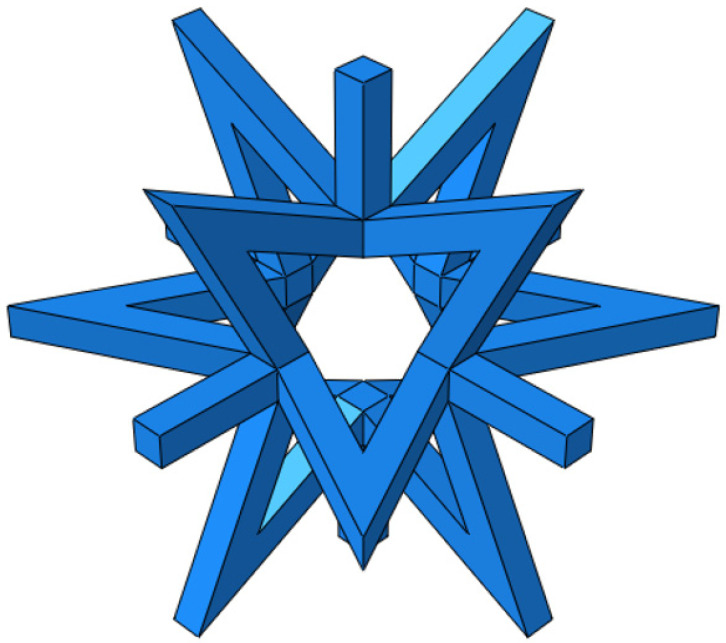
Negative Poisson’s ratio cellular structure.

**Figure 3:**
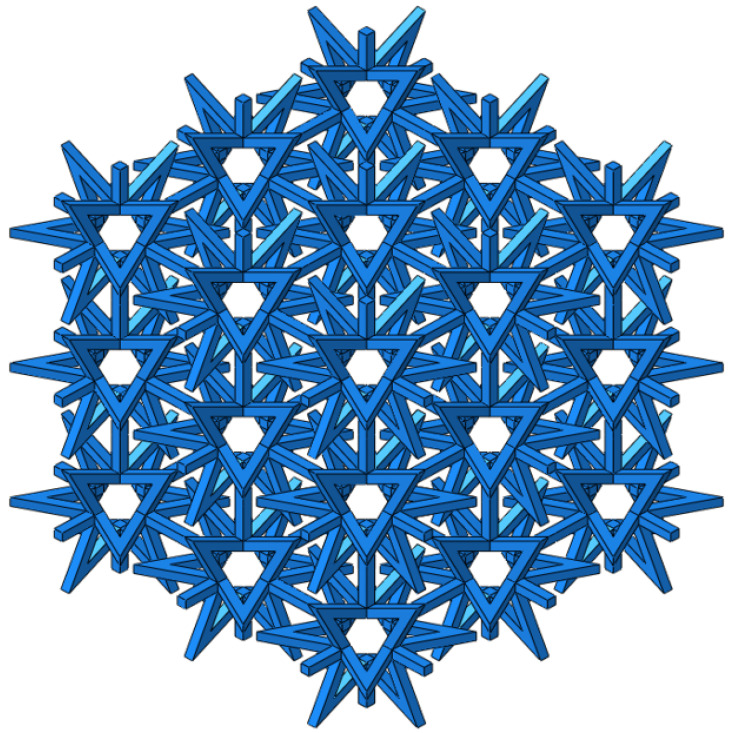
Negative Poisson’s ratio composite structure.

The deformation mechanism of structures with a positive Poisson’s ratio involves shear and twist, whereas structures with a negative Poisson’s ratio deform through concave and rotational mechanisms. Figure 1 displays an octagonal two-dimensional cell with negative Poisson’s ratio properties, which exhibits concave behavior during deformation. When a structure is under tension, it may exhibit a negative Poisson’s ratio effect by expanding along the plane perpendicular to the direction of the applied force. In the case of a star-shaped structure, the angle of the arm is denoted by *θ*, the length of the arm is denoted by *α*, the thickness of the arm is denoted by *t*, and the total length in the X and Y directions are denoted by *Lx* and *Ly*, respectively. A star-like three-dimensional negative Poisson’s ratio cell was formed by rotating the octagonal two-dimensional negative Poisson’s ratio cell-element 90° around its central axis, as illustrated in Figure 2. Figure 3 shows a composite structure based on this three-dimensional cell, exhibiting a negative Poisson’s ratio.

Based on the theory of homogenization, it is assumed that the meso-structure is both periodic and uniformly distributed. This allows the overall structure to be described as a combination of the uniform macro-structure and the non-uniform, periodically arranged mesostructure, providing a comprehensive representation of the system. The full-size method and representative volume element method are both commonly utilized to determine the mechanical properties of honeycomb structures. The full-size method involves analyzing the entire structure at the macroscopic level, which requires a significant amount of computation. On the other hand, the representative volume element method involves analyzing a smaller, representative section of the structure in order to make predictions about the larger structure. The representative volume element (RVE) method aims to determine the equivalent mechanical properties of a honeycomb structure by selecting an appropriate RVE and applying periodic boundary conditions to the fundamental unit cell. In this study, the RVE method is combined with the investigation of a star-shaped honeycomb structure that exhibits a negative Poisson’s ratio. The finite element homogenization method proposed by Sun C and Vaidya, R. [24] is employed as a direct method based on the RVE approach, which involves applying suitable periodic boundary and loading conditions to the structure to derive relevant governing equations. By performing calculations and analyses, it is possible to accurately predict the equivalent elastic constants of periodic structures.

### 2.2. Numerical Simulation of Three-Dimensional Star-Shaped Negative Poisson’s Ratio Structures

#### 2.2.1. Numerical Simulation of Cellular Elements

To characterize the geometric properties of a three-dimensional star-shaped cell with a negative Poisson’s ratio, the following parameters were measured: thickness (*t*) = 2 mm, length (*α*) = 11.18 mm, and angle between the arms (*θ*) = 36.87°. The selected material for testing was a resin material (*ABS-M30*) with an elastic modulus of 2560 MPa and a Poisson’s ratio of 0.3. The unit type used was an 8-node hexahedral linear reduced integral element (*C3D8 R element*). To simulate the boundary conditions, a fixed constraint was applied to the bottom of the structure, and a tension load was applied to the top surface, which was coupled to a point. The displacements at the vertices and sides of the structure were measured and analyzed. The equivalent elastic modulus and Poisson’s ratio were calculated according to Equations (1) and (2).

(1) $$E=\frac{\sigma }{\varepsilon }=\frac{F/S}{\Delta L/L}$$

(2) $$v_{xy}=-\frac{\varepsilon_{y}}{\varepsilon_{x}}=-\frac{\Delta y/y}{\Delta x/x}$$

**Figure 4:**
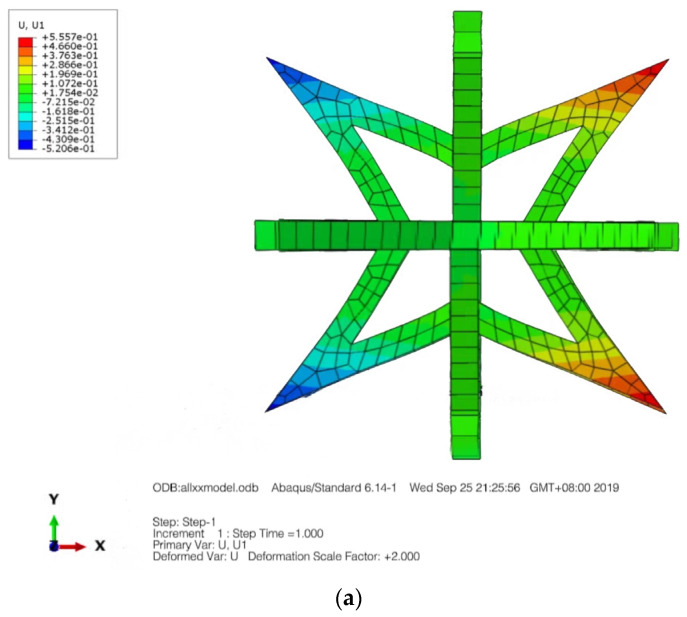

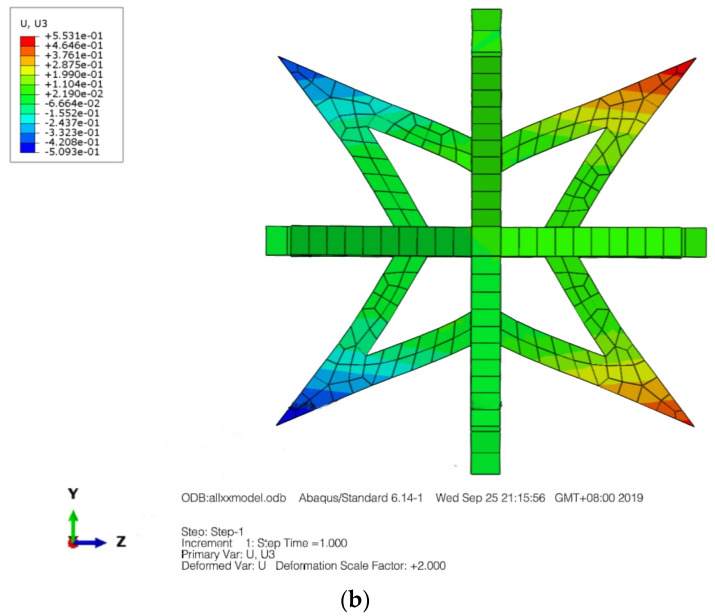
Displacement contour graph. (**a**) X-direction. (**b**) Y-direction.

In the given formula, it appears that *F* represents the applied force, s represents the average cross-sectional area of the rod, *L* is the original length and Δ*L* represents the elongation. Furthermore, *x* and *y* represent the original length of the cell in two directions, while Δ*x* and Δ*y* represent the elongation in two directions. Figure 4 displays a displacement nephogram of a three-dimensional star-shaped cell exhibiting a negative Poisson’s ratio in both the X and Y directions, and this distribution appears to be symmetric.

The star-shaped cell structure can be considered as a slender member, with an equivalent cross-sectional area denoted as s = V/H, where *V* is the cell volume (v = 1327.5 mm^3^) and *h* is the cell height (*H* = 30 mm). The equivalent cross-sectional area is calculated to be 44.25 mm^2^. The structure exhibits symmetry about the X, Y, and Z axes, such that Ex = Ey = EZ.

When a representative volume unit of the three-dimensional star-shaped cell is subjected to an axial tension of 20 n, the resulting elongation in the X direction is denoted as △*x* = 0.557 mm, and similarly, △*L* = 0.557 mm and △*y =* △*z* = 0.557 mm. By substituting these values into Formulas (1) and (2), we obtain the equivalent Poisson’s ratio and equivalent elastic modulus of the three-dimensional star-shaped cell.

#### 2.2.2. Numerical Simulation of Composite Structures

**Figure 5:**
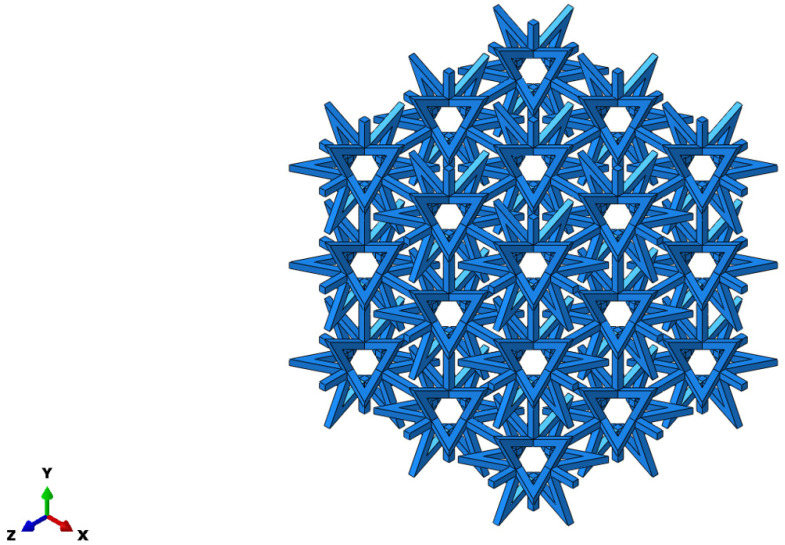
Composite structure.

By comparing different cell arrangements, it was discovered that when three or more cells are combined on one side, the difference in the equivalent elastic modulus and equivalent Poisson’s ratio becomes negligible. In order to enhance the computational efficiency and ensure reliable results, this study selects a composite structure comprising 27 cells, with 3 cells arranged in the X, Y, and Z directions, as depicted in Figure 5.

**Figure 6:**
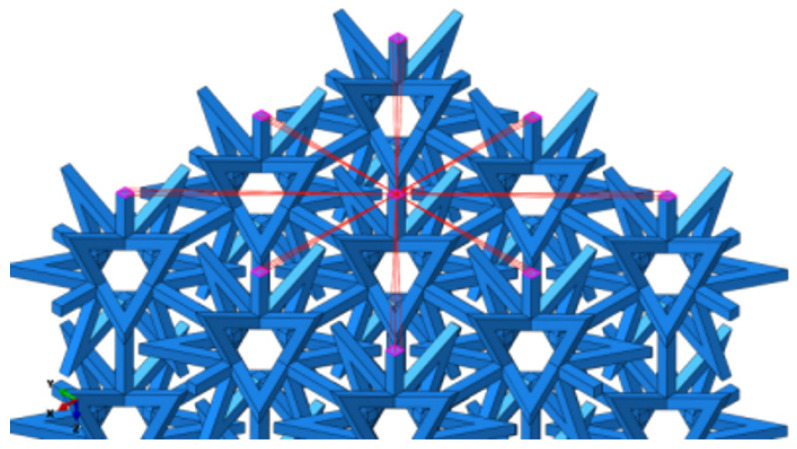
Coupling loading.

**Figure 7:**
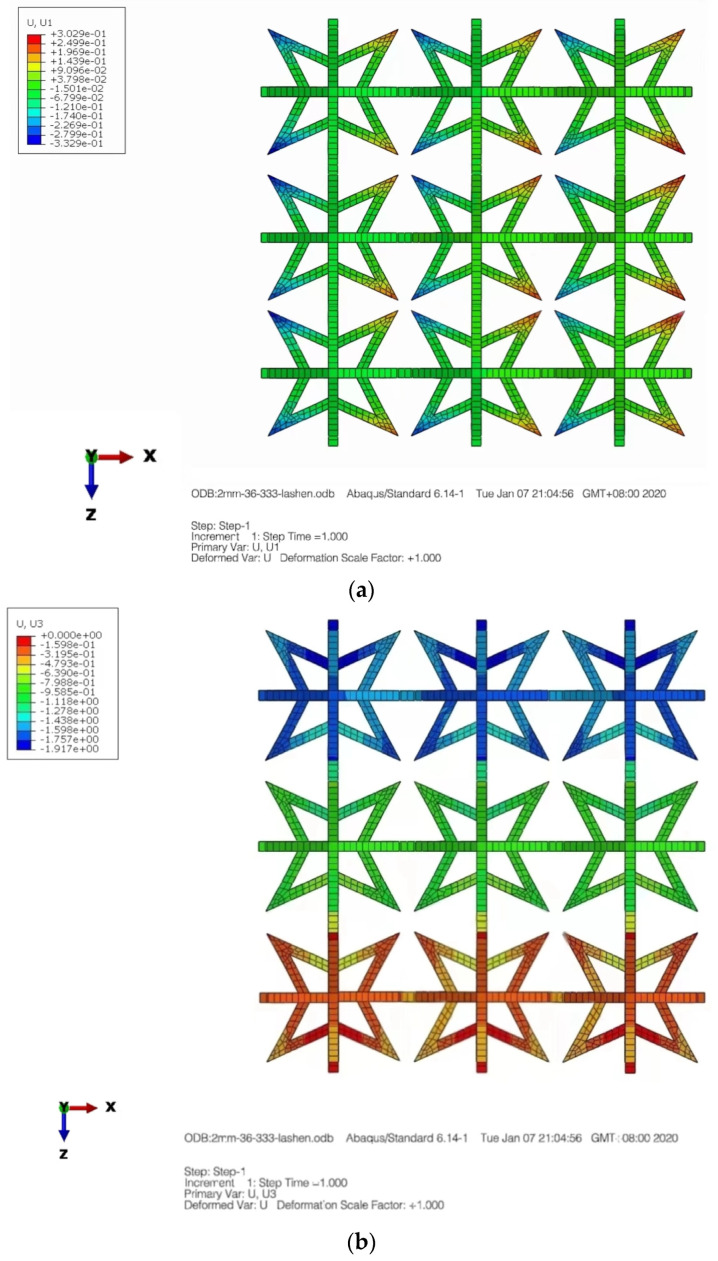
Displacement contour graph in the X and Z directions. (**a**) X-direction displacement nephogram. (**b**) Z-direction displacement nephogram.

The boundary conditions for the composite structure are illustrated in Figure 6, where the bottom is fixed with constraints, while the top surface is coupled to a point where a load is applied. Displacement nephograms of the structure under tension are shown in Figure 7a,b, depicting displacement in the X and Z directions, respectively.

The equivalent cross-sectional area of the composite structure is *S* = 398.25 mm^2^, *L* = 90 mm. An axial tension of 200 N is applied along the negative direction of the Z axis, resulting in a displacement of ∆*Z =* ∆*L* = 1.917 mm and ∆*Y* = 0.09 mm. By substituting these values into Equations (1) and (2), the equivalent Poisson’s ratio and equivalent elastic modulus of the three-dimensional stellate cell can be obtained.

#### 2.2.3. Bending Simulation of Composite Structures

**Figure 8:**
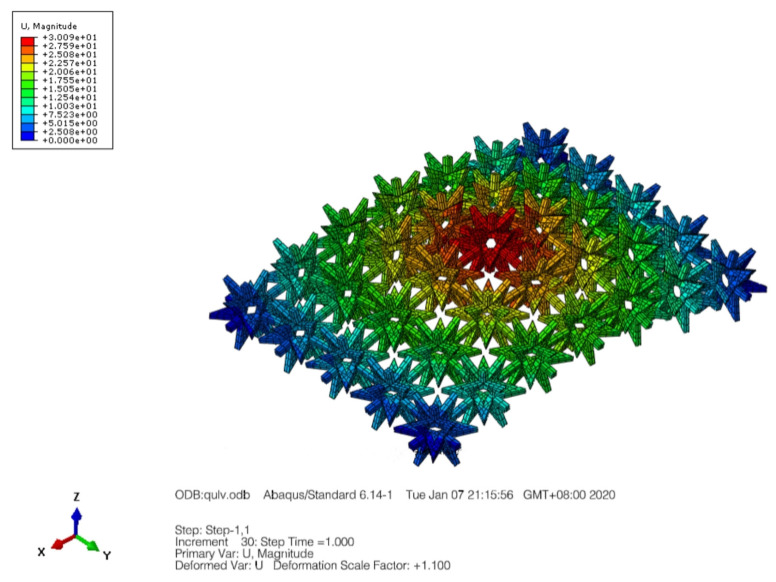
Deformation of the star-shaped honeycomb structure by the bending moment outside the surface.

**Figure 9:**
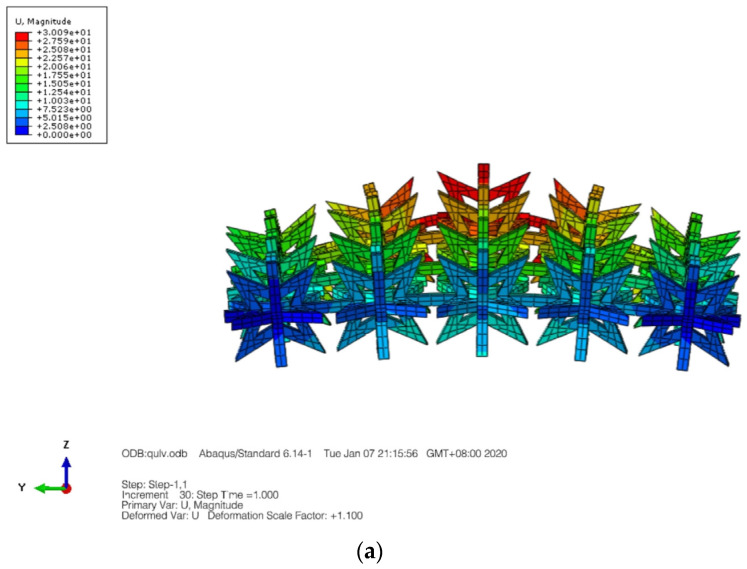

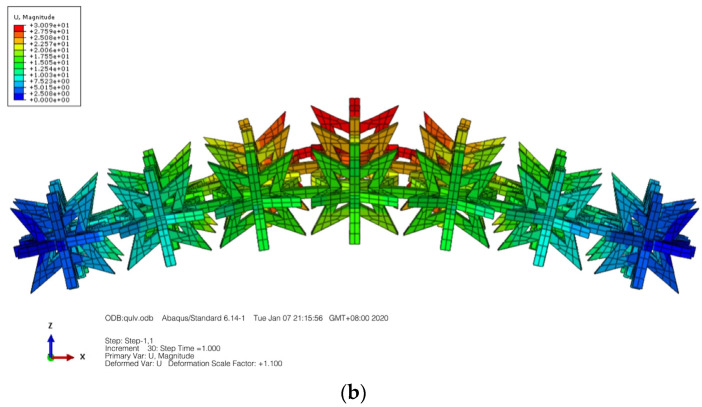
Deformation of the 3D negative Poisson’s ratio composite structure in the yz and xz planes. (**a**) The YZ plane structural deformation. (**b**) The XZ plane structural deformation.

The material exhibiting a negative Poisson’s ratio demonstrates hyperbolic behavior when subjected to out-of-plane loading, resulting in a characteristic arch-shaped middle bulge structure. In this study, we conducted bending simulations on a three-dimensional star-shaped composite structure with a negative Poisson’s ratio. Our model utilized a 6 × 6 × 1 plane structure and applied a torsional load, along with a 200 N/mm out-of-plane bending moment at the structure’s boundary. Figure 8 and Figure 9 illustrate the deformation observed in our simulations. By fitting the coordinates of the midpoint at the top of the cell in both the YZ and XZ planes, we obtained two curve equations. These equations were then used to calculate the maximum deformation point’s curvature by applying them to the curvature calculation formula as in Equation (3).

(3) $$K=\left|\frac{d\alpha }{ds}\right|=\frac{y^{\prime \prime }}{{\left(1+y^{\prime 2}\right)}^{3/2}}$$

By substituting the data, we calculated the deformation curvature K1 of the YZ plane structure to be 0.0049 and the deformation curvature K2 of the XZ plane structure to be 0.0056. This calculation confirms the double curvature characteristics of the star-shaped composite structure with a negative Poisson’s ratio.

### 2.3. Model Test of a New Three-Dimensional Star-Shaped Negative Poisson’s Ratio Structure

**Figure 10:**
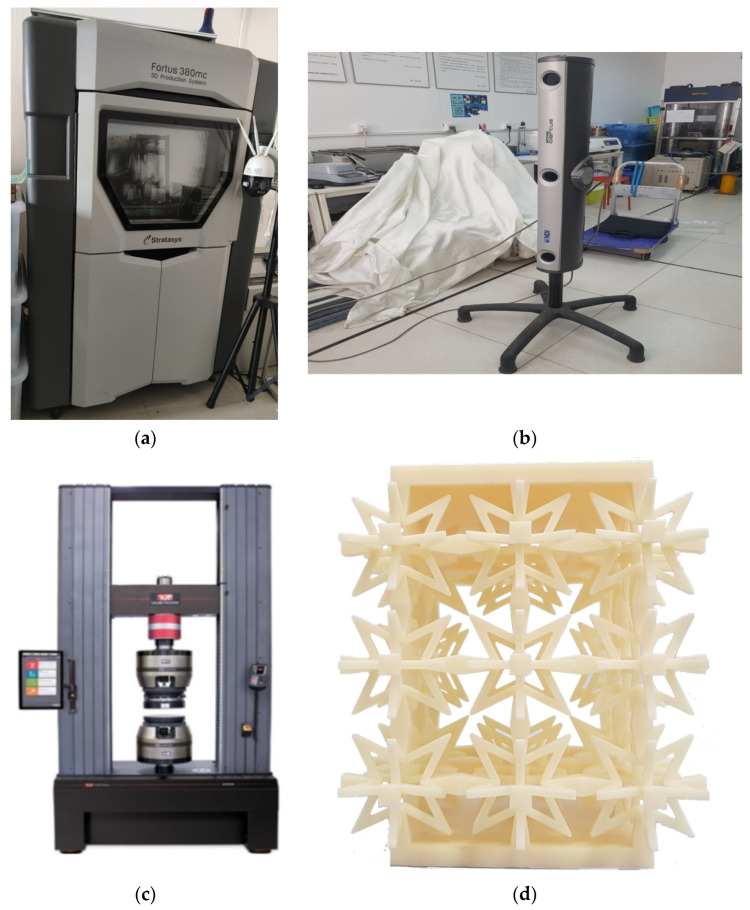
The experiment apparatus and model. (**a**) Fortus 3D printer. (**b**) Optotrak Certus motion capture system. (**c**) Instron universal testing machine. (**d**) Star-shaped negative Poisson’s ratio test model.

**Figure 11:**
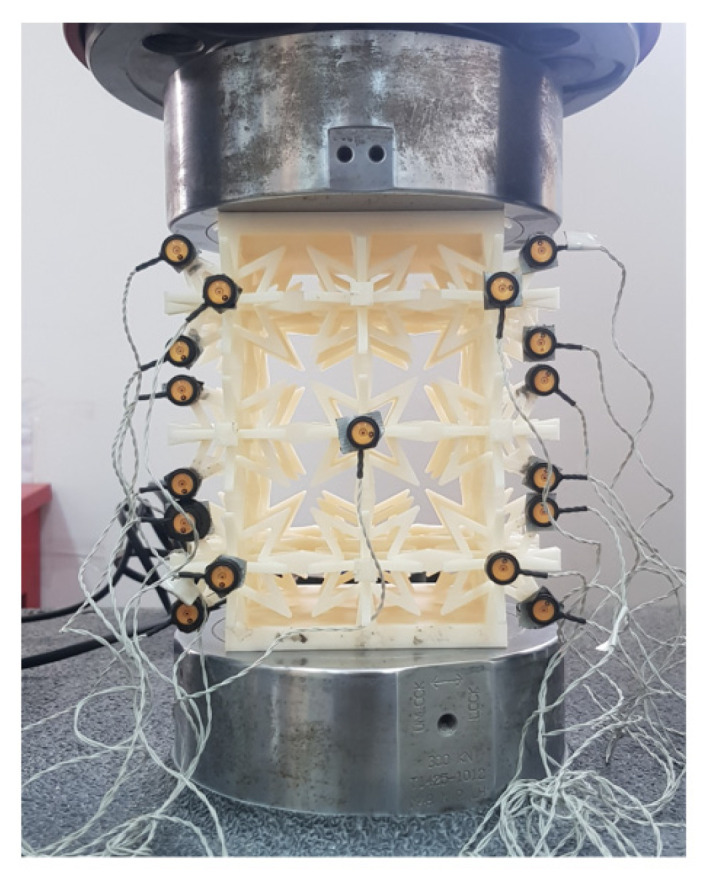
Arrangement of measuring points.

**Figure 12:**
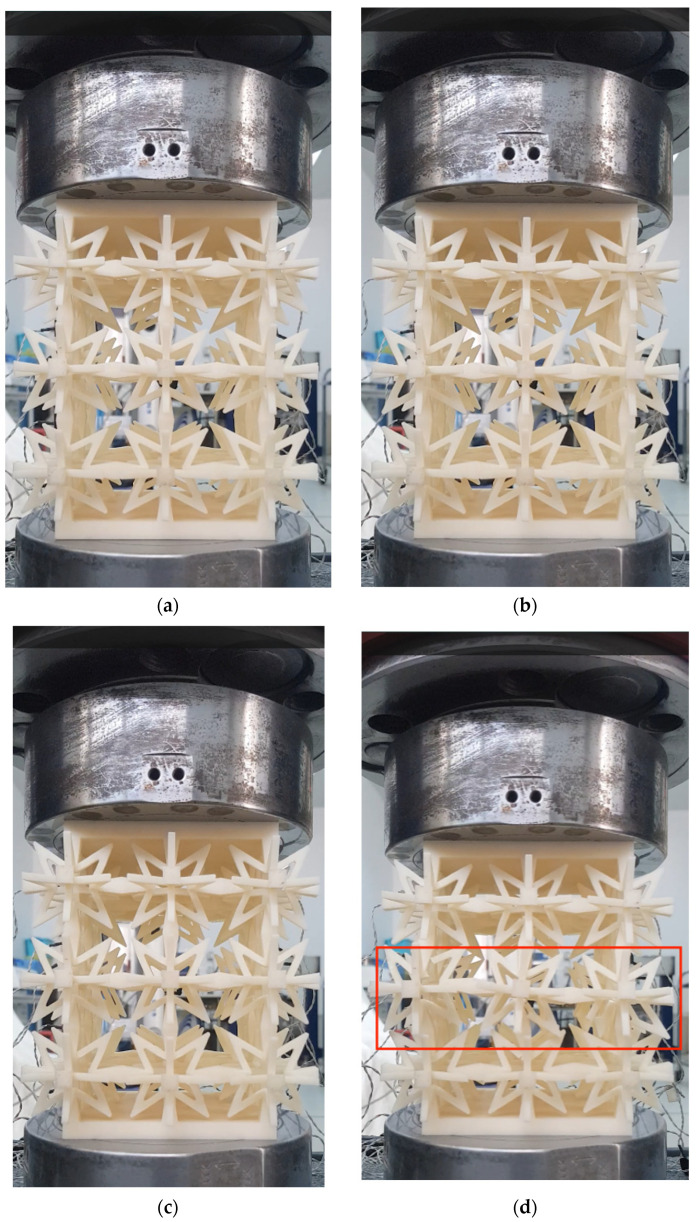
Component compression experiment process. (**a**) No compression state. (**b**) Compression 3 mm. (**c**) Compression 6 mm. (**d**) Component fracture.

The Fortus 380 mc 3D printer from Stratasys, a US-based company, was used to produce the test model using ABS-M30 as the production material. In order to accurately measure small structural deformations, the Optotrak Certus dynamic capture system, manufactured by NDI, a Canadian company, was utilized to collect test data at a frequency of 50 Hz. The test was conducted in accordance with the national standard GB/T1452-1987 “Test Method for Horizontal Tensile Strength of Non-Metallic Sandwich Structures”, using an INSTRON universal testing machine with a loading rate of 6 mm/min. By collecting the data, the equivalent elastic modulus and Poisson’s ratio were calculated and compared with the results of finite element calculations. The test device and test model are depicted in Figure 10, while the layout of measuring points is illustrated in Figure 11. The compression test procedure for the component is illustrated in Figure 12.

## 3. Results and Discussion

### 3.1. Equivalent Modulus of Elasticity and Equivalent Poisson’s Ratio

Table 1 shows the equivalent Poisson’s ratio and equivalent elastic modulus of the three-dimensional star-shaped cell element, and Table 2 shows the equivalent elastic modulus and Poisson’s ratio of the composite structure. The spatial displacement data measured by the compression test are brought into Equations (1) and (2) to obtain the equivalent elastic modulus and equivalent Poisson’s ratio of the test shown in Table 3.

Upon comparison of Table 1 and Table 2, it can be observed that the equivalent Poisson’s ratio differs by 4.1%, while the equivalent elastic modulus *E* differs by 3.1%. These results suggest that the cell element and the composite structure exhibit similar mechanical deformation characteristics.

The equivalent elastic modulus of the star-shaped composite structure Ey(Ex) obtained from the experiment differs by 17.0% from the value calculated by the finite element method, while the Poisson’s ratio shows a difference of 6.12%. Notably, the error in the equivalent elastic modulus is relatively large. This is due to the expansion of the connection section during the model printing process, which was performed to ensure the connection’s reliability. As a result, the mechanical performance of the structure slightly increased, but the numerical value is still valid. The data presented above demonstrate that the finite element simulation results are in good agreement with the experimental results, confirming the accuracy of the numerical model and verifying the excellent negative Poisson’s ratio effect of the newly designed three-dimensional star-shaped cell and the negative Poisson’s ratio composite structure based on it. Moving forward, a key parameter analysis of this composite structure with a negative Poisson’s ratio will be conducted to gain a more systematic and in-depth understanding of this new type of three-dimensional negative Poisson’s ratio.

### 3.2. Three-Dimensional Star-Shaped Component Force–Displacement Curve

**Figure 13:**
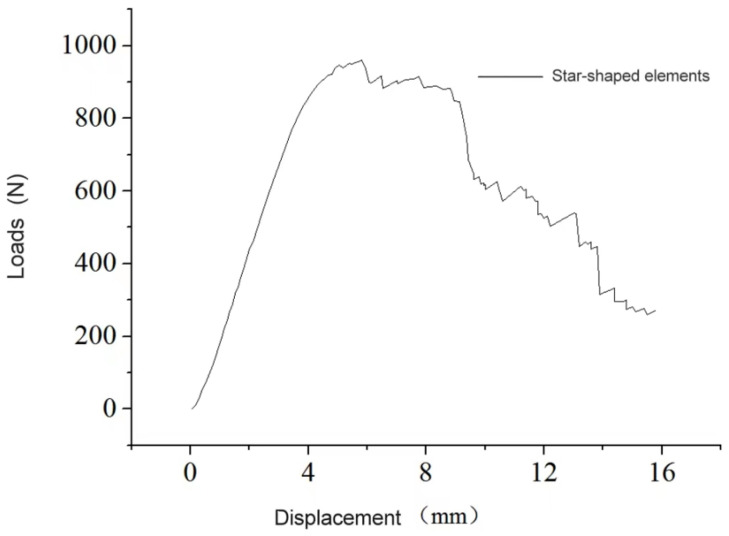
Three-dimensional star-shaped component force–displacement curve.

The force–displacement relationship is depicted in Figure 13. Initially, during the displacement range of 0–4 mm, the curve exhibits a linear upward trend. Upon reaching 4–6 mm, the component enters the plastic stage, and the slope of the curve gradually decreases. At a displacement of 5.85 mm, the first connecting rod fractures, resulting in a drop in bearing capacity from 960 N to 897 N. As the displacement increases, the load increases slightly to 917 N. However, at this point, the second connecting rod fractures, leading to a significant decline in the bearing capacity. Subsequently, several connecting rod fractures occur successively. The bearing capacity curve of the component follows the following pattern: it initially rises, but with each fracture of the connecting rod, the bearing capacity decreases sharply until the component is ultimately destroyed, and the test comes to an end.

### 3.3. Key Parameter Analysis

#### 3.3.1. Effect of Structural Dimensions on the Mechanical Properties of the Star-Shaped Negative Poisson’s Ratio Composite Structures

The independent variables selected for the study were the arm angle, denoted as *θ*, and the arm thickness, denoted as *t*. The objective was to investigate the impact of different combinations of these variables on the equivalent elastic modulus and equivalent Poisson’s ratio.

**Figure 14:**
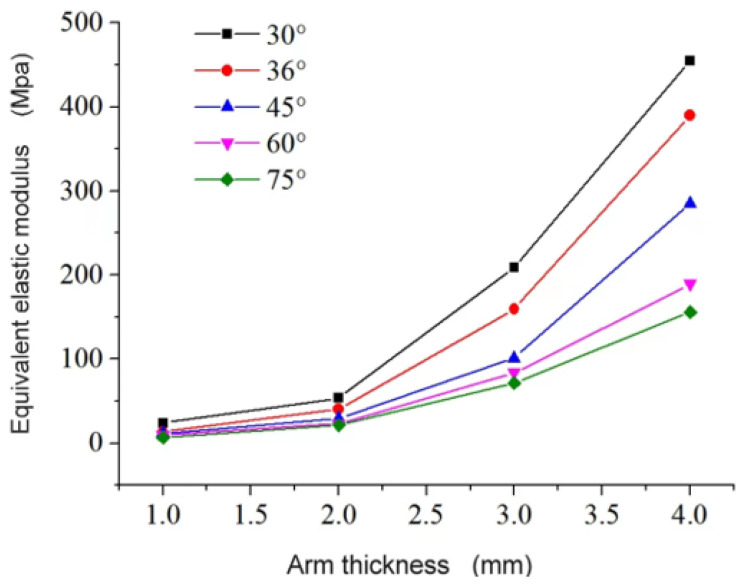
The equivalent elastic modulus of different honeycomb wall thicknesses varies with θ angle.

**Figure 15:**
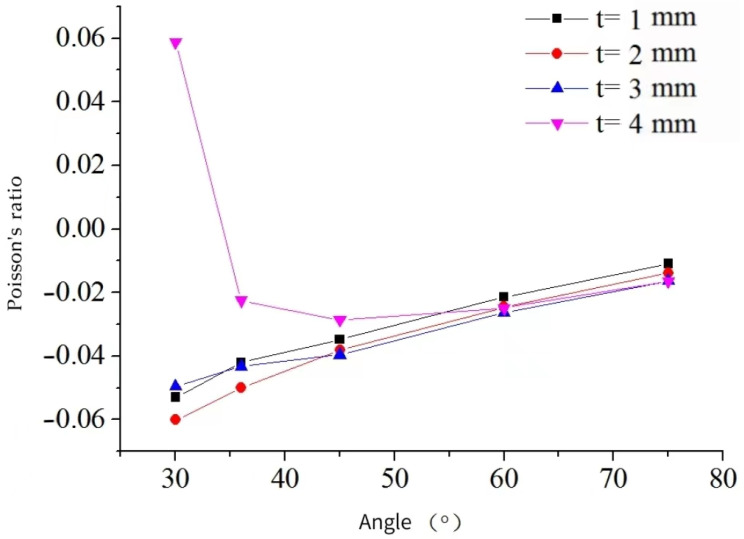
Poisson’s ratio varies with the θ angle for different honeycomb wall thicknesses.

Figure 14 illustrates that the equivalent elastic modulus increases as *t* increases while *θ* remains constant and decreases as *θ* increases while *t* remains constant. Meanwhile, Figure 15 demonstrates the impact of varying *θ* on the Poisson’s ratio for a three-dimensional negative Poisson’s ratio composite structure with different arm thicknesses. Specifically, when the arm thickness is constant at *t* = 1, 2, or 3 mm, the Poisson’s ratio approaches 0 as *θ* increases. However, for *t* = 4 mm, the Poisson’s ratio first decreases from +0.058 to −0.029, then increases to −0.021, due to the fully solid structure at *θ* = 30° which cannot exhibit compression shrinkage characteristics, resulting in a positive Poisson’s ratio. These findings indicate that the mechanical properties of star-shaped negative Poisson composite structures are significantly influenced by their size.

#### 3.3.2. Effect of Material Properties on the Mechanical Properties of the Star-Shaped Negative Poisson’s Ratio Composite Structures

The study investigates the effect of altering the material properties on the equivalent elastic modulus and equivalent Poisson’s ratio of a star-shaped negative Poisson composite structure, with a fixed cell length, width, and height of 30 mm, and a unified selection of *θ* = 36° and *t* = 3 mm. The relevant properties of the material in the numerical model are modified, and the impact on the structure’s mechanical properties is analyzed. The relevant data are presented in Table 4, Table 5 and Table 6.

Table 4 shows the effect of the material modulus of elasticity, based on 160,000 MPa; every 6000 MPa is a magnitude, a total of 11 models are established, and 200 N vertical force is applied. The results show that: the equivalent modulus of elasticity of the honeycomb structure increases with the increase in the material modulus of elasticity; the equivalent Poisson’s ratio of the star-shaped negative Poisson composite structure with different moduli of elasticity is −0.052 which indicates the material modulus of elasticity has almost no effect on the equivalent Poisson’s ratio of the structure; under the current conditions, the equivalent elastic modulus of the star-shaped negative Poisson composite structure with different elastic moduli is about 4.49% of the material elastic modulus.

Table 5 presents the impact of the material’s Poisson’s ratio on the equivalent elastic modulus and the equivalent Poisson’s ratio of the star-shaped negative Poisson composite structure. The results demonstrate that the elastic modulus of the star-shaped structure is approximately 4.5% of the material’s elastic modulus, and the equivalent elastic modulus has a positive association with the material’s Poisson’s ratio. Specifically, for every 0.05 increase in the material’s Poisson’s ratio, the equivalent elastic modulus rises by 1.5%, and the equivalent elastic modulus of the structure grows with the increase in the material’s Poisson’s ratio. Moreover, the negative Poisson’s ratio effect is more evident when the material’s Poisson’s ratio is larger.

Table 6 illustrates the influence of different material types on the equivalent elastic modulus and equivalent Poisson’s ratio of the star-shaped negative Poisson composite structure. To ensure minimal deformation of the structure, the applied load for rubber material and nylon material is reduced to 1 N and 20 N, respectively, while for the other materials, it is 200 N. The analysis indicates that the equivalent elastic modulus of the star-shaped negative Poisson’s composite structure is around 4.5% of the material’s elastic modulus, and rubber displays the best negative Poisson’s ratio effect. Regarding metal materials, the copper alloy exhibits as the best effect, but there is little difference among them, ranging from −0.058 to −0.050.

## 4. Conclusions

Based on an octagonal two-dimensional negative Poisson’s cell, a three-dimensional star-shaped negative Poisson’s cell and a three-dimensional star-shaped negative Poisson’s composite structure are proposed. Through numerical simulation, model test, and parameter analysis, the mechanical characteristics of a three-dimensional star-shaped negative Poisson composite structure are discussed:

(1) The negative Poisson’s ratio effect and hyperbolic properties of the three-dimensional star-shaped negative Poisson’s ratio cytomatrix and three-dimensional star-shaped negative Poisson’s composite structure were verified, and the analysis showed that the equivalent Poisson’s ratio of the cytomatrix and the composite structure differed by 4.1%, The difference in the equivalent elastic modulus is 3.1%, which indicates that the cytogenic analysis can reflect the mechanical characteristics of the composite structure to a certain extent.

(2) The experimental values of the equivalent elastic modulus are similar to the finite element calculation results, which verifies the correctness of the model and proves that the newly designed 3D star-shaped negative Poisson’s ratio cell element and composite structure have a good negative Poisson’s effect.

(3) Parametric analysis shows that: the main factor affecting the equivalent Poisson’s ratio and equivalent elastic modulus of the structure is the form of the structure; the equivalent elastic modulus is proportional to *t* and inversely proportional to *θ*; the Poisson’s ratio approaches 0 as the angle of *θ* increases; the equivalent elastic modulus is positively correlated with the material Poisson’s ratio and the equivalent elastic modulus increases by 1.5% for every 0.05 increase in the material Poisson’s ratio.

(4) For eight different real materials, the negative Poisson’s ratio effect does not vary much, with the effect for all of them being between −0.058 to −0.050. The best effect is for rubber. For metallic materials, the effect of the copper alloy is relatively the best.

## Figures and Tables

**Table 1:** Equivalent elastic modulus and equivalent Poisson’s ratio of the cell.

Parameter	Numerical Value
Ex(Ey,Ez) MPa	24.34
υxy (υyz,υxz)	−0.048

**Table 2:** The equivalent elastic modulus and Poisson’s ratio of the composite structure.

Parameter	Finite Element Calculation
Ex(Ey,Ez) MPa	23.58
υxy(υyz,υxz)	−0.046

**Table 3:** Experimental test results.

Parameter	Test Value
Ey(Ex) MPa	28.42
υxy	−0.049

**Table 4:** The parameters of the star-shaped negative Poisson’s ratio structure with different elastic moduli.

Elastic Modulus of Material (MPa)	Poisson Ratio	Equivalent Elastic Modulus (MPa)	Equivalent Poisson’s Ratio
160,000	0.3	7182.16	−0.052
166,000	0.3	7451.02	−0.052
172,000	0.3	7720.82	−0.052
178,000	0.3	7990.16	−0.052
184,000	0.3	8259.49	−0.052
190,000	0.3	8528.82	−0.052
196,000	0.3	8798.12	−0.052
202,000	0.3	9067.49	−0.052
208,000	0.3	9336.82	−0.052
214,000	0.3	9606.15	−0.052
220,000	0.3	9875.48	−0.052

**Table 5:** Various parameters of the star-shaped negative Poisson’s ratio structure with different Poisson’s ratios.

Material Modulus (MPa)	Poisson Ratio	Equivalent Elastic Modulus (MPa)	Equivalent Poisson’s Ratio
200,000	0.1	8536.97	−0.0485
200,000	0.15	8633.74	−0.0492
200,000	0.2	8738.98	−0.0501
200,000	0.25	8853.32	−0.0511
200,000	0.3	8977.71	−0.0522
200,000	0.35	9113.17	−0.0535
200,000	0.4	9261.19	−0.0550
200,000	0.45	9423.77	−0.0567

**Table 6:** The parameters of the star-shaped negative Poisson’s ratio structure with different materials.

Material	Elastic Modulus of Material	Poisson Ratio	Equivalent Elastic Modulus	Equivalent Poisson’s Ratio
rubber	6.1	0.49	0.29	−0.0582
nylon	1000	0.3	44.89	−0.0522
HT250 cast iron	66,178.1	0.27	2945.53	−0.0515
7075-T6 aluminum alloy	72,000	0.33	3260.72	−0.0530
Titanium alloy	110,000	0.3	4937.74	−0.0522
Copper alloy	110,000	0.37	5043.92	−0.0541
KTB380-12 cast iron	120,000	0.31	5402.32	−0.0524
Q235	210,000	0.27	9346.90	−0.0515

## Data Availability

The data used to support the findings of this study have not been made available because they also form part of an ongoing study.
